# Modeling CO_2_ solubility in water using gradient boosting and light gradient boosting machine

**DOI:** 10.1038/s41598-024-63159-9

**Published:** 2024-06-12

**Authors:** Atena Mahmoudzadeh, Behnam Amiri-Ramsheh, Saeid Atashrouz, Ali Abedi, Meftah Ali Abuswer, Mehdi Ostadhassan, Ahmad Mohaddespour, Abdolhossein Hemmati-Sarapardeh

**Affiliations:** 1 Department of Petroleum Engineering, Shahid Bahonar University of Kerman, Kerman, Iran; 2 Department of Chemical Engineering, Amirkabir University of Technology (Tehran Polytechnic), Tehran, Iran; 3 College of Engineering and Technology, American University of the Middle East, 54200 Egaila, Kuwait; 4 Institute of Geosciences, Marine and Land Geomechanics and Geotectonics, Christian-Albrechts-Universität, 24118 Kiel, Germany; 5 Department of Chemical Engineering, McGill University, Montreal, QC H3A 0C5 Canada

**Keywords:** CO_2_ solubility in pure water, Intelligent model, GBoost, LightGBM, Chemical engineering, Engineering

## Abstract

The growing application of carbon dioxide (CO_2_) in various environmental and energy fields, including carbon capture and storage (CCS) and several CO_2_-based enhanced oil recovery (EOR) techniques, highlights the importance of studying the phase equilibria of this gas with water. Therefore, accurate prediction of CO_2_ solubility in water becomes an important thermodynamic property. This study focused on developing two powerful intelligent models, namely gradient boosting (GBoost) and light gradient boosting machine (LightGBM) that predict CO_2_ solubility in water with high accuracy. The results revealed the outperformance of the GBoost model with root mean square error (RMSE) and determination coefficient (R^2^) of 0.137 mol/kg and 0.9976, respectively. The trend analysis demonstrated that the developed models were highly capable of detecting the physical trend of CO_2_ solubility in water across various pressure and temperature ranges. Moreover, the Leverage technique was employed to identify suspected data points as well as the applicability domain of the proposed models. The results showed that less than 5% of the data points were detected as outliers representing the large applicability domain of intelligent models. The outcome of this research provided insight into the potential of intelligent models in predicting solubility of CO_2_ in pure water.

## Introduction

Phase equilibria calculations of carbon dioxide (CO_2_) solubility in water holds significant importance in a variety of research areas due to the rising worldwide application of this gas for a broad range of purposes. CO_2_ is one of the greenhouse gases that cause major ecological issues, including global warming and climate change effects. Over the last few years, carbon capture and utilization (CCU) and carbon capture and storage (CCS) techniques have attracted significant interest from researchers as effective greenhouse gas mitigating strategies^1,2^. Underground geological formations, such as depleted oil and gas reservoirs, deep saline aquifers, and salt domes have been identified as reliable sites for the CCS process^3^. Depleted gas and oil reservoirs have been widely regarded as a highly promising choice, primarily due to their large storage capacity and the vast number of available data attained from exploration and production operations^4–6^. The oil industry also takes the advantage of CO_2_ injection into oil and gas reservoirs as a promising enhanced oil recovery (EOR) method^7,8^. Moreover, the combination of water and CO_2_ injection, which is known as water-alternating-gas (WAG), is considered a successful technique because of its ability to control gas mobility and reduce the viscous fingering phenomenon^9,10^. As seen in many fields, the interactions between water and CO_2_ play an important role and the dissolution of CO_2_ could affect the ultimate storage capacity in CCS^11,12^ and the performance of EOR methods. From an environmental perspective, it could also pose a leakage risk by contaminating underground water^13–16^.

Accordingly, CO_2_ dissolution in water has been intensively investigated using a variety of experimental techniques^17–20^. One of the earliest studies that examined the solubility of CO_2_ in water at sequestration conditions was conducted by Wiebe and Gaddy^21^ at temperatures between 323 and 373 K and pressures up to almost 71 MPa. According to the literature, the maximum pressure at which the solubility of CO_2_ gas in water has been experimentally measured is 350 MPa which was reported in the studies of Todheide & Franck^22^ and Takenouchi and Kennedy^23^. In 1999, Dhima et al. measured the CO_2_ solubility in pure water at 344.25 K and pressures up to 100 MPa^24^. Ahmadi and Chapoy^25^ utilized a high-pressure setup to examine the CO_2_ solubility in several salt solutions at temperatures between 300 and 424 K and pressures up to 41 MPa. They conducted experiments with deionized water under equal conditions to verify the validity of their method. Wang et al.^26^ conducted experiments to determine the solubility of CO_2_ in water under high pressure and temperature conditions. They investigated the effect of the vapor phase of H_2_O. A summary of literature experimental research os presented in Table 1, comprising the respective year of study, applied pressure and temperature ranges, and the employed equipment.

**Table 1:** Literature experiments studies on the determination of CO_2_ solubility in water.

Year of study	Temperature (K)	Pressure (MPa)	Utilized equipment	References
1939	323–373	2.53–70.93	CO_2_ compressed and solubility bomb	^21^
1940	285.15–313.15	2.53–50.66	CO_2_ compressed and solubility bomb	^17^
1963	304.15–647.15	20–350	Cylindrical high-pressure vessel	^22^
1964	383.15–623.15	10–150	Autoclave in an electric furnace	^23^
1992	288.15–313.15	5.17–20.27	Recirculation equipment	^19^
1999	344.25	10–100	Flash in equilibrium cell	^24^
2018	300.95–423.48	1.292–40.349	Pneumatic rocking system	^25^
2021	313.15–473.15	0.5–200	Cylindrical equilibrium vessel	^26^

CO_2_ solubility in water can also be estimated by thermodynamic modeling, which is more cost-effective than experimental measurements. In recent years, the potential of theoretical models to effectively represent influential characteristics such as pressure, temperature, and electrolyte concentrations has attracted the interest of researchers attempting to develop models for different systems^27^. Duan and Sun^28^ proposed a model to estimate CO_2_ solubility in water and aqueous NaCl solutions using an extensive databank containing about 1500 data points at temperatures ranging from 273 to 533 K and pressures between 0 and 2000 bar. Spycher et al.^29^ utilized a calculating approach based on determining new Redlich-Kwong Equation of State (EoS) parameters^19^ for mutual solubility between CO_2_ and water, using the CO_2_ solubility data obtained at 285–373 K and up to 60 MPa. Yan et al.^30^ measured CO_2_ solubility in water and NaCl brine in three temperatures of 232, 373, and 413 K and a pressure range of 5 to 40 MPa. By comparing experimental data with Søreide and Whitson EoS model^31^, they indicated that a modification in the model by refitting the interaction parameter between water and CO_2_ in the aqueous phase could lead to an acceptable solubility prediction. Recently, statistical associating fluid theory (SAFT) EoSs have also been applied to CO_2_–electrolyte–water solutions^27,32,33^. Yan and Chen^34^ created a model for CO_2_ solubility in NaCl solution employing a Perturbed-Chain SAFT (PC-SAFT) EoS with a maximum temperature and pressure of 473.15 K and 150 MPa. They determined Henry's constant by fitting the experimental CO_2_ solubility in pure water at the same pressure and temperature range. Moreover, multiple thermodynamic models of CO_2_ solubility have been constructed based on various pressures, temperatures, and water salinity conditions^25,35,36^. Table 2 provides an informative summary of theoretical models found in the literature. It includes information such as the year of study, the pressure and temperature ranges that were examined, the techniques used for prediction, and the error.

**Table 2:** Literature theoretical models on the calculation of CO_2_ solubility in water.

Year of study	Temperature (K)	Pressure (Mpa)	Applied Technique	Error	References
2003	273–533	0–200	Thermodynamic model	AAD% = 7	^28^
2003	285–373	0–600	Modified Redlich-Kwong EoS	AAD% = 5	^29^
2005	274.15–473.15	2.2–60	SAFT1-RPM EOS	AAD% = 0.28	^33^
2010	273.15–473.15	0.0075–150	PC-SAFT EoS	Residual rootmean square error = 4.75	^34^
2011	232–413	5–40	Modified Søreide and Whitson EoS	AAD% = 8.49	^30^
2018	300–424	0–40	Simplified cubic plus association EoS (sCPA-EoS)	AAD% = 6.88	^25^
2022	298.15–573.15	0–5.2	Electrolyte perturbed hard sphere chain (e-PHSC) EoS	AAD% = 2.28	^27^

In recent years, artificial intelligence (AI) has garnered significant attention from researchers and has become a potent tool for predicting tasks in a variety of fields^37–41^. AI has been widely applied in the oil industry owing to its advantages over costly and time-consuming experimental procedures and sophisticated thermodynamic models^42^. AI was utilized in the assessment of a water-alternating-CO_2_ process^37^ for determining the minimum miscibility pressure (MMP) of CO_2_^38^ and predicting oil recovery in CO_2_-EOR approaches^39,40^. A considerable number of studies have been carried out in the field of environmental research, specifically in the area of CCS, which have provided valuable insights into the potential of CCS as a feasible approach to mitigate climate change. These studies have focused on predicting the efficiency of CO_2_ storage^41^ and assessing the features of coal as an approach to carbon sequestration^42,43^.

Ghasemian et al.^44^ developed an artificial neural network (ANN) to estimate the solubility of CO_2_ in water based on 105 data points from their experiments conducted at pressure and temperature ranges of 0.1–1 MPa and 278.15–348.15 K, respectively. They noticed that the ANN model outperformed the EoSs in CO_2_ solubility prediction with an absolute average deviation (AAD) of 4%. Hemmati-Sarapardeh et al.^45^ investigated the efficiency of four machine learning (ML) techniques, namely, radial basis function (RBF), multilayer perceptron (MLP), gene expression programming (GEP), and least-squares support vector machine (LSSVM) models, to estimate CO_2_ solubility in pure water at high pressures and temperatures. The results showed that the highest level of accuracy was achieved with the LSSVM model optimized by the firefly optimization algorithm (FFA) with a root mean square error (RMSE) of 0.3261. Khoshraftar and Ghaemi utilized ANN and response surface methodology (RSM) to predict the solubility of CO_2_ in water. Their model development was based on 240 measurements that were conducted within a pressure range of 0.5–200 MPa and a temperature between 313.15 and 473.15 K. The efficiency of MLP and RBF models were compared using an ANN technique. All the developed models accurately predicted solubility, although the RBF and MLP models exhibited the highest performance^46^.

Jeon and Lee^47^ developed an ANN based on 2406 data points to predict CO_2_ solubility in pure water and single-salt aqueous solutions. In order to train the model, 80% of the data bank was used, and the remaining 20% of the data points of solubility in the complex or multi-component solutions were utilized for validation and testing. They stated that the developed ANN model could be extrapolated to predict CO_2_ solubility in multi-component salt solutions. Over the past years, several investigations have been conducted on the prediction of pure and impure CO_2_ solubility^48^ in brine solutions containing different components with the aid of AI^49–51^. Furthermore, research has been carried out to predict the solubility of CO_2_ in the non-aqueous phase. Certain EOR techniques employ the injection of miscible CO_2_ into the oil in order to enhance oil mobility by lowering the viscosity of the oil, the IFT, and oil swelling. Rostami et al. developed a gene expression programming (GEP) model for predicting the solubility of CO_2_ in both dead and live oil, utilizing 106 and 74 data points, respectively. The error analysis revealed that the QEP-based model predicts the solubility of CO_2_ in both dead oil and living oil accurately, with correlation coefficients (R^2^) of 0.9860 and 0.9844 for each^52^. Prior studies that have explored the application of AI to predict the solubility of gases in predicting the solubility of gases in different types of aqueous solutions and non-aqueous phase are summarized in Table 3. This table presents data regarding the year of the study, the applied AI technique, the kind of gas, the solution type, and the temperature and pressure ranges.

**Table 3:** Literature AI models on the prediction of gas solubility in aqueous and non-aqueous phase.

Year of study	Temperature (K)	pressure (Mpa)	Type of gas	Type of aqueous and non-aqueous phase	Number of data points	utilized AI technique	References
2012	278.15–348.15	0.1–1	CO_2_	Water	105	ANN (genetic algorithm (GA))	^44^
2017	291.48–413.15	0.5–32.76	CO_2_	Dead oil/live oil	180	GEP-based model	^52^
2018	291.48–413.15	0.5–32.76	CO_2_	Dead oil/live oil	180	LSSVM	^53^
2019	273.15–723.15	0.098–1400	CO_2_	NaCl brine	570	MLP, RBFNN	^50^
2019	286.91–433.16	0.00651–88.558	CO_2_	Salt solutions	608	LSSVM, ANN, particle optimization swarm- adaptive network-based fuzzy inference system (PSO-ANFIS), GA-ANFIS	^51^
2020	197.6–623.15	0.0003–350	CO_2_	Water	785	MLP, RBF, LSSVM, GEP	^45^
2021	273.15–473.65	0.092–71.231	CO_2_	Water/various salt solutions	2406	ANN	^47^
2022	283.1–373.55	0.000028–30.1	N_2_O	Ionic liquids	801	Belief network (DBN), extreme gradient boosting (XGB), Cat-Boost, extreme learning machine (ELM),	^54^
2022	273.15–318.15	1.51–21.74	CO_2_–N_2_	Water/brine solutions	289	Decision Tree (DT), GB-DT, AdaBoost-DT, AdaBoost-SVR, GB-SVR	^48^
2022	298–373	0.1–20.26	CO_2_	NaCl brine	164	XGB, MLP, K-nearest neighbor (KNN), GA	^49^
2023	313.15–473.15	0.5–200	CO_2_	Water	240	MLP, RBF	^46^

The purpose of this study is to develop intelligent models that accurately predict CO_2_ solubility in pure water. For this, a large data bank with 785 data points containing the values of pressure, temperature, and CO_2_ solubility in water is gathered. Then, two powerful intelligent models, namely gradient boosting (GBoost) and light gradient boosting machine (LightGBM) are implemented to provide predictions for the CO_2_ solubility as a function of temperature and pressure. Various statistical and graphical methods are employed to assess the validity and precision of the developed intelligent models. In addition, a trend analysis is undertaken to verify the developed models’ ability to detect physical trends. Lastly, the validity of the data bank and application domain of both models is examined by the Leverage method. As depicted in Table 3, although there are valuable artificial models for forecasting CO_2_ solubility in aqueous solutions, blending data points of CO_2_ solubility in pure water, single salt solutions, and diverse brines have been implemented in most predicted models. This study comprises an extensive database covering a broad range of temperature and pressures with reference to CO_2_ solubility in water. This vast data collection resulted in a well-trained AI model with high accuracy. Both development models offered in this study demonstrate a high level of accuracy with a minimum determination coefficient of 0.995, indicating the reliability of the models. When employing EOR techniques or carbon storage technologies, we encounter a complex scenario involving the combination of gases in a water solution with varying salt levels and components. These validated and precise AI models can be employed in future research to assess complex systems, such as gas mixes and aqueous solutions of varying salinity levels. This not only improves the reliability of these models but also creates new opportunities for enhancing the effectiveness of these operations.

## Methodology

### Data gathering

In order to develop intelligent models, a trustworthy and broad data set was collected from various sources^17,19,20,22,24,55–87^. Each of the 785 data points in the database contained values for temperature, pressure, and CO_2_ solubility in pure water. Temperature, pressure were regarded as the models’ inputs, while solubility was specified as the models’ output parameter. These data points have already been used by Hemmati-Sarapardeh et al.^45^. The data set was randomly partitioned into 80% and 20% subsets for model training and testing, respectively. Table 4 describes the statistical features of the data bank.

**Table 4:** Statistical overview of the dataset in this study.

Parameter	Type	Mean	Minimum	Maximum	Standard deviation
Temperature (K)	Input	365.91	197.6	623.15	97.59
Pressure (bar)	Input	229.27	0.003	3500	437.47
CO_2_ solubility (mol/kg)	Output	1.68	0.00136	19.72	2.77

### Modeling techniques

#### Gradient boosting (GBoost)

**Figure 1:**
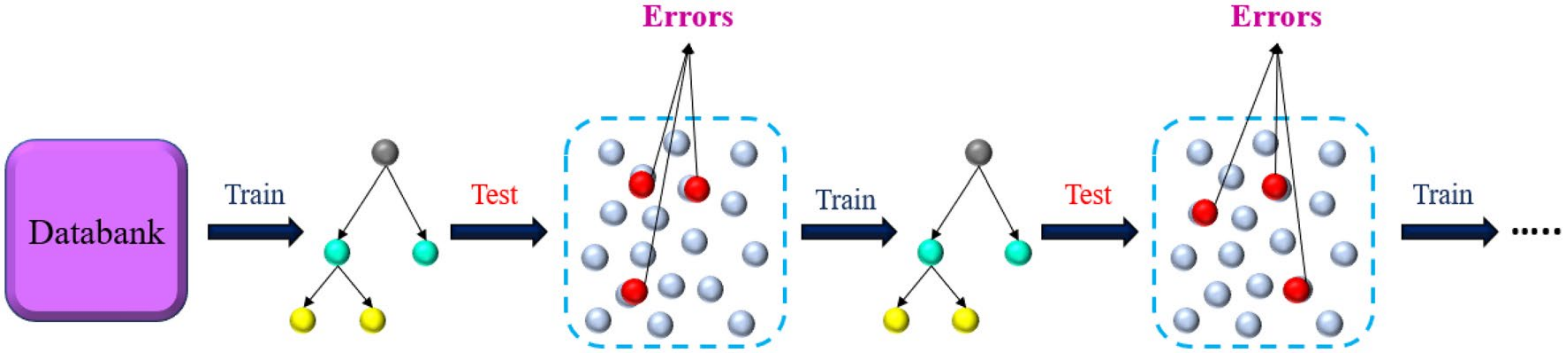
The schematic image of the GB algorithm.

Gradient boosting (GB) is a kind of ensemble supervised tree-based machine learning (ML) approaches that can be utilized for both regression and classification issues^88–90^. It is called an ensemble because the ultimate model's prediction is produced based on various single models’ (decision trees) predictions^88^. GB, which is portrayed in Fig. 1, is an iterative accumulation of sequentially organized tree-based models of weak learners or predictors that are converted to powerful learners^91,92^. Commonly, boosting techniques combine weak predictors into a powerful one in an iterative path to minimize the loss function^89^. This loss function is minimized similarly to an ANN in which weights are tuned^93^.

To achieve this purpose, it is recommended to choose a function $h(x,{\theta }_{t})$ to be the most parallel to the negative gradient ${({g}_{t}\left({x}_{i}\right))}_{i=1}^{N}$. By selecting an iterative approach, we can defeat challenge posed by the prediction variables. The function ${g}_{t}\left(x\right)$ for every experimental data is calculated as below^94^:

1 $${g}_{t}\left(x\right)={E}_{y} {\left[\frac{\partial \Psi (y,f(x))}{\partial f(x)}| x\right]}_{f\left(x\right)={\widehat{f}}^{t-1}(x)}$$

To permit the replacement of a hard optimizing problem, one can easily choose the new function increment to be the most matched with ${-g}_{t}\left(x\right)$ utilizing classic least-squares optimization as follows^89^:

2 $$\left({\rho }_{t},{\theta }_{t}\right)={argmin}_{\rho ,\theta }\sum_{i=1}^{N}{\left[-{g}_{t}\left({x}_{i}\right)+\rho h\left({x}_{i},\theta \right)\right]}^{2}$$

The following stages show a general optimizing procedure of the GBoost^94^:***

Initializing the ${\widehat{f}}_{0}$ as a constant;

Calculating the negative gradient of ${-g}_{t}\left(x\right)$;

Conforming a next base-learner function $h(x,{\theta }_{t})$;

Recognizing the optimal gradient descent step-size ${\rho }_{t}$ as:

3 $${\rho }_{t}={argmin}_{\rho }\sum_{i=1}^{N}\Psi \left[{y}_{i},{\widehat{f}}_{t-1}\left({x}_{i}\right)+\rho h\left({x}_{i},{\theta }_{t}\right)\right]$$

Updating the model's prediction:

4 $${\widehat{f}}_{t}\leftarrow {\widehat{f}}_{t-1}+{\rho }_{t}h\left(x,{\theta }_{t}\right)$$

This approach involves the base-learner phase, which consists of a single neuron and the loss function employed is the standard squared error. Through the process of training of the model, the optimum structure is earned.

#### Light gradient boosting machine (LightGBM)

**Figure 2:**
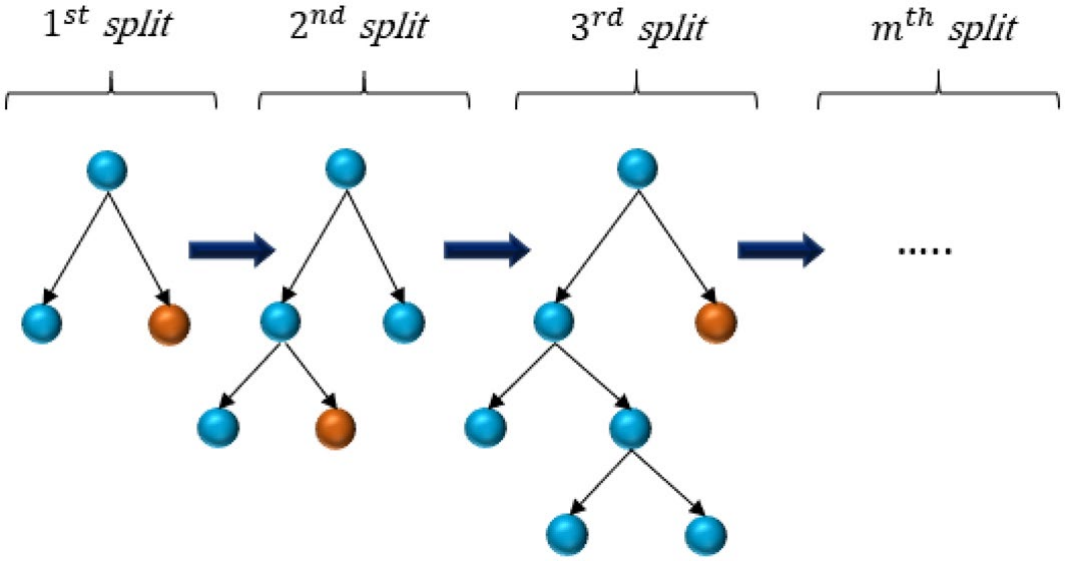
Schematic illustration of the LightGBM.

LightGBM is a gradient-supervised technique based on decision trees and the idea of boosting algorithms^95^. LightGBM technique, which includes several decision trees, is applicable in various ML tasks like regression, classification, and ranking^96–98^. Each LightGBM technique employs a powerful learning framework to produce prediction values^99^. Its principal differences from other tree-based models are that it accelerates the training stage by applying histogram-based techniques, decrease memory consumption and it uses a leaf-wise growth strategy with depth constraints^95^. Figure 2 illutrates a schematic image of the LightGBM. The training process of the LightGBM is determined by the subsequent formula:

5 $$X={\left\{\left({x}_{j},{y}_{j}\right)\right\}}_{j=1}^{N}$$

Next, ${\widehat{f}}_{(x)}$ will forecast by minimizing the loss function $L$ as^95^:

6 $$L\left(y,f\left(x\right)\right): \widehat{f}\left(x\right)=argmin {E}_{y,x}.L\left(y,f\left(x\right)\right)$$

Eventually, the training stage of every regression tree can be indicated as ${W}_{q(x)} , q \in \left\{\text{1,2},3,\dots ,N\right\}$; where *W* denotes a weight term of every leaf node, *q* shows utilized decision rules in each tree, and *N* indicates the number of leaves in a tree^100^. Thus, by the employing of Newton's method for recognizing objective function, the training outcome of every stage is tuned by the following equation:

7 $${G}_{t}\cong \sum_{i=1}^{N}L[{y}_{i}, {F}_{t-1}\left({x}_{i}\right)+{f}_{t}({x}_{i})]$$

## Results and discussion

### Model's development

This research focused on proposing two intelligent models that accurately predict the solubility of CO_2_ in pure water. The models were designed to predict the target variable as a function of temperature and pressure. The established models were trained with 628 data points (80% of the data bank) and tested with 157 data points (20% of the data bank). The accuracy of the intelligent models in this study compared to the EoSs is impressive. All statistical and graphical error analyses verify this issue. Furthermore, smart techniques require less input parameter information than EoSs. In our current research, we only utilized pressure and temperature to predict the solubility of CO_2_ in pure water. In contrast, many EoSs necessitate additional properties. Moreover, the application of EoSs typically consumes a significant amount of time.

For the tuning process, various ranges of hyperparameters were tested to find the optimal value of each hyperparameter. Table 5 shows the optimum values of the GBoost and LightGBM hyperparameters, separately. Max depth refers a maximum depth of the tree. Learning rate determines the step size at each sequential iteration. Min sample split and Min sample leaf show the minimum number of samples needed for splitting an internal node and to form a leaf node, respectively. Also, N estimators represents the number of trees in the forest.

**Table 5:** Optimum values of hyperparameters of the models.

Model	Parameter	Optimum value
GBoost	Max depth	3
	Min sample leaf	3
	Min sample split	2
	Learning rate	0.332
	N estimators	260
LightGBM	Max depth	4
	Min data in leaf	2
	Learning rate	0.5
	Number of leaves	6

### Performance evaluation

To analyze the performance of the developed models, multiple statistical and graphical evaluations were utilized. This research implemented the root mean square error (RMSE) and the correlation coefficient (R^2^) for statistical evaluation. Equations (8) and (9) provide the mathematical formulations of these criteria.

8 $$RMSE= {\left(\frac{1}{n} \sum_{i=1}^{n}{\left({y}_{i}^{exp}-{y}_{i}^{cal}\right)}^{2}\right)}^{0.5}$$

9 $${R}^{2}=1-\frac{{\sum_{i=1}^{n}\left({y}_{i}^{exp}-{y}_{i}^{cal}\right)}^{2}}{{\sum_{i=1}^{n}\left({y}_{i}^{exp}-\overline{y }\right)}^{2}}$$

where ${y}_{i}^{exp}$ is the experimentally determined solubility of CO_2_ in pure water and ${y}_{i}^{cal}$ is the value of solubility predicted by the models. Also, y represents the mean value of the measured data points. As Table 6 reflects, the two developed models showed strong agreement with experimental data. Although error indices indicate the great accuracy of both models, the GBoost model outperformed the LightGBM algorithm slightly.

**Table 6:** Statistical error factors of established models.

Models	RMSE			R^2^		
	Train	Test	All	Train	Test	All
GBoost	0.107	0.220	0.137	0.9985	0.9934	0.9976
LightGBM	0.161	0.285	0.192	0.9963	0.9921	0.9952

**Figure 3:**
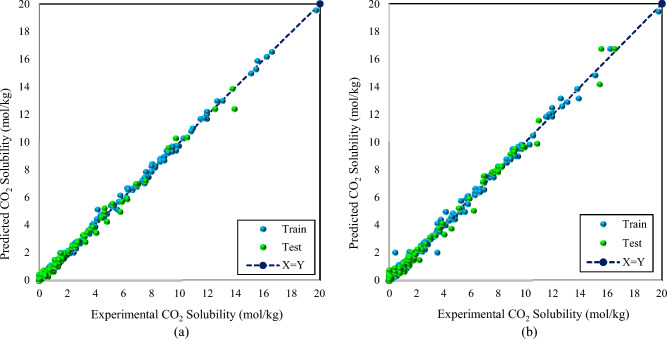
Cross plots for the developed intelligent models, (**a**) GBoost and (**b**) LightGBM.

Graphical assessments were also used to compare the reliability of the models. For this purpose, various kinds of plots, comprising cross-plots, error distribution plots, and cumulative frequency were plotted. Cross plots are one of the visual assessments that compare the predicted data with experimental measurements. The concentration of data near the Y = X line indicates the accuracy of the developed model. Figure 3 shows the cross plots of the GBoost and LightGBM models. As shown, the dense compactness of train and test data points was seen around the unit slope line. While the points were more distributed close to the Y = X line in the cross plot of the LightGBM model, the GBoost approach demonstrated a stronger concentration around this line.

**Figure 4:**
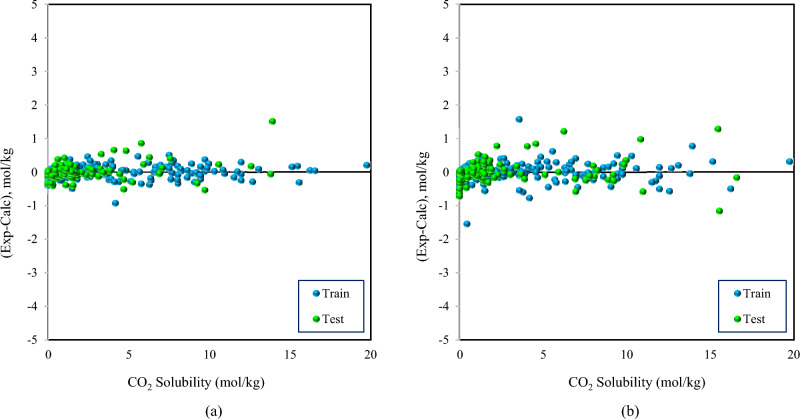
Error distribution plots for (**a**) GBoost and (**b**) LightGBM models.

Figure 4 demonstrates the error distribution diagrams of the established models, where the error is defined as the deviation of predicted data of the solubility from the experimental values. In this diagram, more aggregation around the zero line implies a more accurate model. The plots revealed that the majority of points were located close to the zero-error line and confirmed the accurate predictions. While the GBoost model has an accurate performance over the CO_2_ solubility range, a slight deviation was observed in the LightGBM model in a few points, especially in high solubility values, indicating that there was a minor relative error at these values.

### Group error analysis

**Figure 5:**
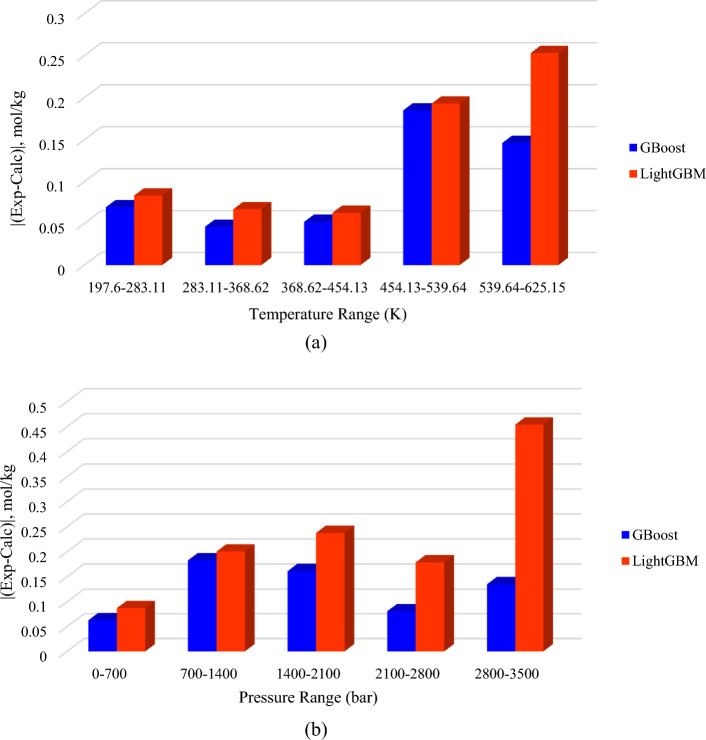
Group error plots of the developed models at different ranges of (**a**) temperature and (**b**) pressure.

Group error diagrams were used to assess the efficiency of the established models in various pressure and temperature ranges. Figure 5 depicts group error plots of the GBoost and LightGBM models in five equal temperature and pressure ranges. As Fig. 5a shows, both models demonstrated higher precision at temperatures below 454.13 K. It should be noted that the GBoost model demonstrated higher reliability than LightGBM throughout all temperature ranges. The effect of pressure on the efficiency of the model was shown in Fig. 5b. As shown, both models provided more accurate predictions at pressures lower than 700 bar. While GBoost demonstrated relatively consistent accuracy at all ranges, increasing the pressure reduced the efficiency of LightGBM.

### Cumulative frequency

**Figure 6:**
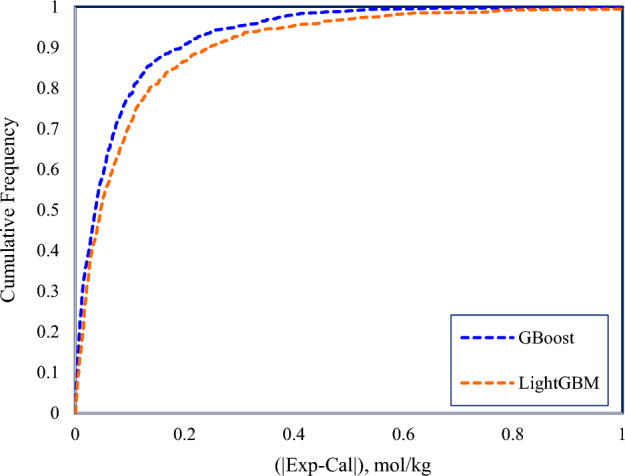
Cumulative frequency of two proposed models for predicting CO_2_ solubility in water.

The cumulative frequency plot is an interactive visualization technique for evaluating the reliability of intelligent models. In these charts, the higher a model is positioned, the more accurate its predictions are. The cumulative frequency curves of the GBoost and LightGBM are illustrated in Fig. 6. The closeness of the two models to the vertical axis signifies that these models predicted the majority of data points with a low error. Considering that the error is defined as the absolute difference between measured experimental and predicted data points, 95% of data predicted by the GBoost model have an error of less than 0.29 mol/kg. By contrast, LightGBM reported 93% of the data at this error value. Both developed models demonstrated an acceptable level of certainty in predicting the solubility of CO_2_, whilst GBoost showed relatively higher accuracy.

### Sensitivity analysis

**Figure 7:**
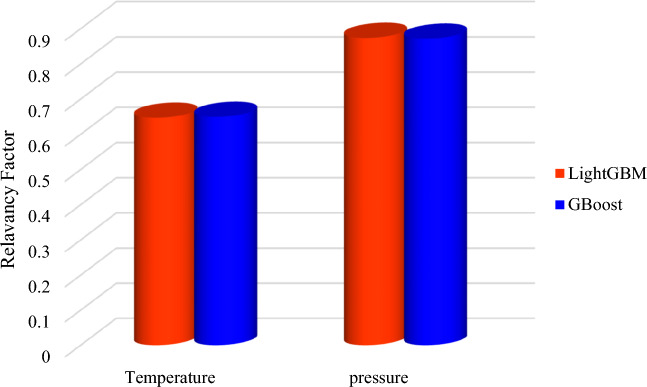
The relative impact of the temperature and pressure on the predicted CO_2_ solubility.

One of the sensitivity approaches employed to measure the effect of input parameters on the output is the relevancy factor. This factor is employed to determine the correlation between dependent and independent variables, and is defined below^101^:

10 $$r\left({I}_{k}, y\right)\frac{\sum_{i=1}^{n}{(I}_{i}^{k}-{I}_{ave}^{k})\left({O}_{i}-{O}_{ave}\right)}{\sqrt{\sum_{i=1}^{n}{\left({I}_{i}^{k}-{I}_{ave}^{k}\right)}^{2}\sum_{i=1}^{n}{\left({O}_{i}-{O}_{ave}\right)}^{2}}}$$

where $I$ and $O$ symbolize the the input and output variables, respectively. As stated earlier, temperature and pressure were considered input parameters of the developed models for predicting CO_2_ solubility in water. ${I}_{i}^{k}$ represents the $i$ th value of $k$th input parameters, ${I}_{ave}^{k}$ stands for the average value of the $k$th input, and ${O}_{ave}$ indicates the average output. The relevancy factor shows a value between − 1 and 1. Where a positive number implies a direct correlation between the input and output variables, while a negative value shows a reverse one. The degree of influence is expressed by the absolute value of $r$ so that the greater the r, the more influence of the input. Figure 7 demonstrates the impact of pressure and temperature on CO_2_ solubility for the GBoost and LightGBM models. As can be seen, the two models reflected relatively the same relevancy factor of input parameters. While a rise of both temperature and pressure would result in an increase in solubility, pressure with a relevancy factor of 0.87 had a stronger impact than temperature with a relevancy factor of almost 0.65.

### Model trend analysis

**Figure 8:**
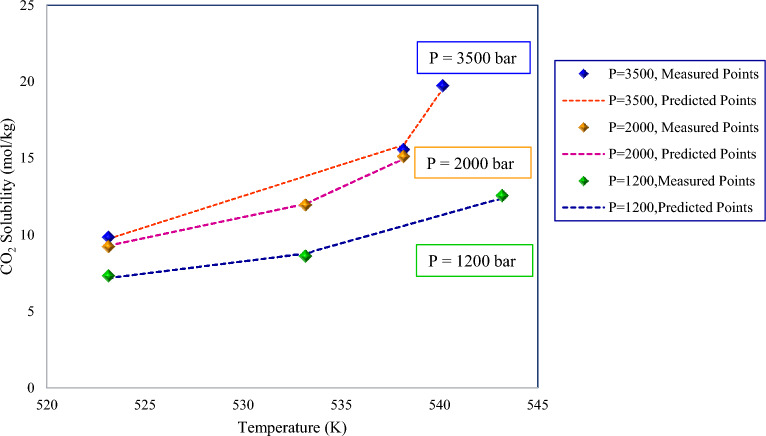
The GBoost model’s predictions and experimentally measured data points of CO_2_ solubility in water at three fixed pressures with temperature variation.

**Figure 9:**
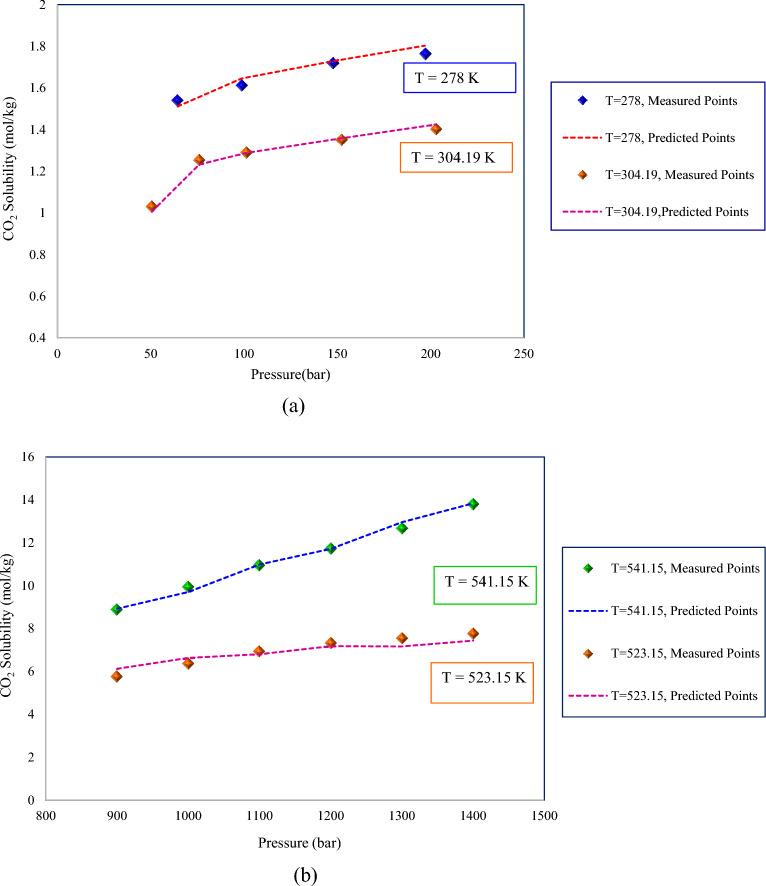
The GBoost model’s predictions and measured data points of CO_2_ solubility in water at fixed temperatures with pressure variation (**a**) for low to medium temperature and pressure (**b**) for high temperature and pressure.

As noted in previous sections, the GBoost approach provided more accurate CO_2_ solubility predictions. To investigate the impact of pressure and temperature on the CO_2_ solubility in pure water and to verify the model's engagement to a physically expected trend, the experimentally measured points were compared to the GBoost model’s predictions with respect to the trends of solubility at various temperatures and pressures by keeping one of them at a constant value. The temperature's impact on solubility at three constant pressures is depicted in Fig. 8. As demonstrated, the predicted points were accurately consistent with the experimental data and illustrated that increasing temperatures increases solubility strength. The solubility line at 3500 bar is positioned above the solubility lines at 2000 bar and 1200 bar in the figure, which illustrates the influence of pressure. In addition, the impact of pressure on solubility is represented in Fig. 9. As illustrated in the figure, a rise in pressure enhanced gas solubility at both high and low temperatures. Pressure rise causes compressibility in the gas phase, which leads to the release of more space for additional gas molecules to be dissolved in the water. In Fig. 9a, the solubility at 278 K was higher than that of 304.19 K. Although, in high-pressure regions, the solubility increased as the temperature rise. In other words, at low and medium pressures, heating the liquid or increasing the temperature results in a rise of the molecules’ kinetic energy, leading them to move faster and letting more of them escape the solvent. This indicates that thermal energy overcomes the intermolecular attraction force between carbon dioxide and pure water. However, when gas reaches the supercritical condition, which for CO_2_ is above 304.13 K and 73.97 $bar$ of temperature and pressure, it exhibits a more complicated behavior. As seen in Figs. 8 and 9b demonstrate, CO_2_ tends to behave more as a liquid, showing that both pressure and temperature positively affect solubility^102^. In conclusion, Figs. 8 and 9 confirmed the validity of the GBoost model and revealed that the model accurately captured the phenomenon’s existing physical trend.

### Implementation of the Leverage method

Existing outlier data in a data bank might adversely impact the prediction’s efficiency and applicability of a model. Therefore, the detection of these measured points that differ from the bulk of data is a key step for model development. The Leverage method was employed to detect outliers in this study^103,104^. According to a statistical perspective and a visual evaluation, this technique identifies outlier points. This method sketches the Williams plot based on a $H$ value and standardized residual. The $H$ value, refers to the elements of the Hat matrix, which is computed as below:

11 $$H=X{({X}^{T}X)}^{-1}{X}^{T} , X=N\times k$$

12 $$SR= \frac{{e}_{i}}{{RMSE \left(1-{H}_{ii}\right)}^{0.5}}$$

where $k$ and $N$ indicate the input parameters and number of data points, respectively, and $T$ refers to the transpose. The standardized residual is measured based on the ${e}_{i}$, which is the deviation between predicted and experimental data, root mean square error ($RMSE$), and $H$ vectors. In the Williams plot, valid data points are situated in the area bounded by the Leverage limit (${H}^{*}$), and the suspected limits. This valid zone represents the applicability of the model domain. The mathematical measurement of the Leverage limit is presented in Eq. (13), which corresponds to 0.0115 for the gathered databank. Suspicious limits are defined as a standard residual higher than 3 or less than − 3. In addition, the area with $H>{H}^{*}$ is classified into two areas based on its SR: good high leverage and bad high leverage. The good high leverage, which represented data points that predicted well but were outside the scope of the model's applicability, relates to the data as $-3\le SR\le 3$. The bad high leverage area belongs to points with $SR>3$ or $SR<-3$^105–107^.

13 $${H}^{*}= \frac{3\times (k+1)}{N}$$

where k represents the number of inputs (here two) and N is the total number of data points (here 785).

**Figure 10:**
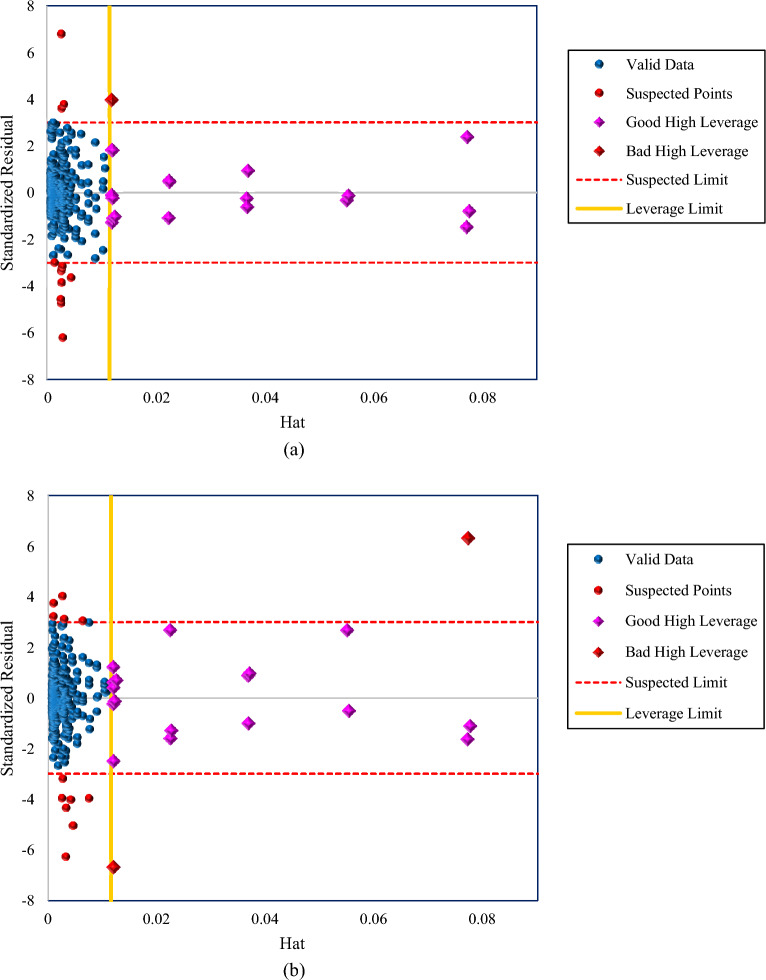
William’s plot for the (**a**) GBoost and (**b**) LightGBM models for identifying suspected and outlier data.

Williams's plot is one of the outlier detection methods for examining the performance of developed models which is widely used in AI application studies^45,108–111^. The William plots of the proposed models for predicting the CO_2_ solubility in water are shown in Fig. 10. As the plots demonstrate, the majority of data points were placed in the valid region, 95.92% of the points for GBoost and 95.67% of them for the LightGBM. For both models, only 2.42% of data exceeded the leverage limit. Overall, as a negligible percentage of data were placed out of the valid zone, the Leverage approach approved the applicability of proposed models and the validity of experimental data points.

## Conclusions

In this study, two tree-based models, GBoost and LightGBM, were developed to predict CO_2_ solubility in pure water based on an extensive data bank including 785 experimental data points collected from diverse sources. Two parameters of pressure and temperature were considered as the input parameters, while solubility was defined as the output.

- In order to validate models, statistical and graphical evaluations were implemented. Multiple validation procedures approved the high precision agreement between experimental and predicted solubility values. The findings indicated the outperformance of the GBoost model with R^2^ and RMSE values of 0.9976 and 0.137 mol/kg, respectively.
- The trend analysis was employed to assess the pressure and temperature effects on the solubility in comparison to the predictions of the model. The trend analysis revealed that the proposed models exhibited the high accuracy in comprehending understanding the physical trend of the problem.
- For outlier detection, the Leverage approach was implemented which demonstrated the validity and reliability of the models on a large portion of data; nevertheless, only a few points were identified as suspected data.
- The findings of this study demonstrated that both developed models could be considered potent and trustworthy tools for predicting the solubility of CO_2_ in water.

## Data Availability

All the data have been collected from literature. We cited all the references of the data in the manuscript. However, the data will be available from the corresponding author on reasonable request.
